# Minimal assessment of index cases: the point of view of the neurologist

**DOI:** 10.1186/1750-1172-10-S1-I11

**Published:** 2015-11-02

**Authors:** Ernst Hund

**Affiliations:** 1 University Heidelberg, Heidelberg, Germany

## 

The first and most important step is to consider TTR-FAP as a diagnosis upon thorough patient history and clinical examination. The further diagnostic process includes the following assessments: clinical examination for polyneuropathic and autonomic signs including temperature and pain sensitivity in the feet. In addition, nerve conduction studies are essential to document large fiber neuropathy. Further on, the patient should be checked for signs of cardiomyopathy and cardiac conduction disorders, since cardiac disease is common in TTR-FAP. Although there will be a characteristic pattern of findings with these examninations, they cannot prove the diagnosis of TTR-FAP. Further investigations include histopathology with demonstration of amyloid by Congo red staining, immunhistochemistry to show that amyloid is composed of TTR, and genetic testing to reveal the specific mutation underlying the disorder.

